# A Talk on Combination Fillings

**Published:** 1893-08

**Authors:** Dwight M. Clapp

**Affiliations:** Boston, Mass.


### A Talk on Combination Fillings.

BY DWIGHT M. CLAPP, D.M.D., BOSTON, MASS.

Read before the Vermont Dental Society, March, 1893.

Six or seven years ago I began filling with amalgam and gold, uniting the gold with the fresh amalgam, and finishing the filling at one sitting, thereby securing the good qualities of each of these materials and making a filling, as it seems to me, theoretically and practically destined to be of real value.

Five years ago I had the honor to accept an invitation from this society to give a clinic at one of its meetings, showing the method of making these combination fillings.

Four years ago last summer I read a paper on the subject before the New York Odontological Society which was published in the *Cosmos*. So far as I know, that paper was the first published giving a method whereby gold could be united with amalgam so as to make a strong union. Since that time much has been written on the subject, and now hardly a dental journal can be read without finding some kind of a combination filling mentioned.

I have recently re-read the article above referred to and find that I would not change what was then written were I to re-write it. I might, indeed, add much to it, but would take nothing from it, and to-day, after all these years of trial and experience, I believe that the filling there described stands unrivaled as a tooth-saver in those cases where it is indicated.

Any one with a fair amount of skill and proper instruction can put a good filling into a cavity that has only favorable conditions. To save teeth that are frail and far-gone and make them do more years of service is what makes us of value to our fellowmen, and it is this that is to endear us to our patients and make one's services absolutely indispensable.

Do not think I flatter myself that I will give you anything new to-day. I come, rather, to hear from your collective experience. What I say, will be said with the hope that it will loosen your tongues, thereby turning into the hopper of discussion your united wisdom and experience, which, when finely ground and carefully bolted will furnish each of us with a sack of knowledge for home consumption.

I suppose, to-day, nearly half of my fillings are a combination of materials, and I believe I am doing better work than ever before.

Now, why may a combination filling be better than one made of a single material? Because it enables us to use each kind of material in that portion of the cavity to which it is best adapted. Few, probably, will deny that amalgam will preserve a tooth better at the cervical portion of an approximal cavity in a molar, or that gold with its greater edge strength is better for the masticating surface. Again, it enables us to make tooth and filling of one mass, or nearly so, so to speak, the filling thus helping to support the tooth, instead of being supported almost entirely by it. For instance, if a cement filling is placed in a cavity the cement adheres to the tooth and if the walls are frail it helps to strengthen them. Now, if, when we have put some sticky cement into a cavity and while it is yet soft we press some amalgam into this sticky cement, we have an adhesion between tooth, cement and amalgam, and the whole makes a solid mass much stronger than if the cavity were filled with a material held in by pressure and the shape of the filling only.

It may be illustrated in this manner: Suppose we are to build a pier for a bridge out of granite blocks. Will it be stronger if the blocks are simply piled one on top of the other, or if each block is united with its neighbor by a layer of the best Portland cement? Therefore, I say, that by combining two, three and sometimes four different materials in one filling, more satisfactory results may often be obtained than by the use of a single material.

You will readily understand that I am not advocating this as an easy way of filling teeth—although often much time and strength are saved—I present it as a good method for saving teeth, not for saving time or skill.

Of all the filling materials we have, cement, probably, has the most good qualities. It is of good color, is adhesive and is a good preserver of teeth, but it lacks the one most essential quality —durability.

Now, if we can fill a considerable portion of large cavities with a material which is capable of taking the place analogous to that of Portland cement in the bridge pier, and protecting it with some other material or materials that have the quality of durability, we are, at least, doing much toward arriving at the ideal filling.

